# Development and evaluation of interpretable machine learning regressors for predicting femoral neck bone mineral density in elderly men using NHANES data

**DOI:** 10.17305/bb.2024.10725

**Published:** 2024-07-05

**Authors:** Wen He, Song Chen, Xianghong Fu, Licong Xu, Jun Xie, Jinxing Wan

**Affiliations:** 1 Reproductive Medicine Center, Quzhou People’s Hospital, The Quzhou Affiliated Hospital of Wenzhou Medical University, Quzhou, China; 2 Department of Orthopedics, Quzhou People’s Hospital, The Quzhou Affiliated Hospital of Wenzhou Medical University, Quzhou, China; 3 Department of Endocrinology, Quzhou People’s Hospital, The Quzhou Affiliated Hospital of Wenzhou Medical University, Quzhou, China

**Keywords:** Femoral neck bone mineral density (FNBMD), osteoporotic femoral neck fractures (OFNFs), National Health and Nutrition Examination Survey (NHANES), machine learning, random forest regressor (RFR)

## Abstract

Osteoporotic femoral neck fractures (OFNFs) pose a significant orthopedic challenge in the elderly population, accounting for up to 40% of all osteoporotic fractures and leading to considerable health deterioration and increased mortality. In addressing the critical need for early identification of osteoporosis through routine screening of femoral neck bone mineral density (FNBMD), this study developed a user-friendly prediction model aimed at men aged 50 years and older, a demographic often overlooked in osteoporosis screening. Utilizing data from the National Health and Nutrition Examination Survey (NHANES), the study involved outlier detection and handling, missing value imputation via the K-nearest neighbor (KNN) algorithm, and data normalization and encoding. The dataset was split into training and test sets with a 7:3 ratio, followed by feature screening through the least absolute shrinkage and selection operator (LASSO) and the Boruta algorithm. Eight different machine learning algorithms were then employed to construct predictive models, with their performance evaluated through a comprehensive metric suite. The random forest regressor (RFR) emerged as the most effective model, characterized by key predictors, such as age, body mass index (BMI), poverty income ratio (PIR), serum calcium, and race, achieving a coefficient of determination (*R*^2^) of 0.218 and maintaining robustness in sensitivity analyses. Notably, excluding race from the model resulted in sustained high performance, underscoring the model’s adaptability. Interpretations using Shapley additive explanations (SHAP) highlighted the influence of each feature on FNBMD. These findings indicate that our predictive model effectively aids in the early detection of osteoporosis, potentially reducing the incidence of OFNFs in this high-risk population.

## Introduction

Osteoporotic femoral neck fractures (OFNFs) represent a prevalent orthopedic challenge among the elderly, comprising up to 40% of all osteoporotic fractures and significantly impairing health while increasing mortality [1–3]. Experts anticipate a 2.7-fold increase in the incidence of such fractures in Eastern Asia, with projections rising from 18,388 cases in 2010 to 50,421 by 2035 [4]. Early detection of individuals with osteoporosis and the implementation of anti-osteoporotic treatments are crucial measures for preventing these fractures [5]. Femoral neck bone mineral density (FNBMD) is recognized as a valuable predictor of OFNFs and is recommended for diagnosing osteoporosis and assessing low bone mass [6]. Although dual-energy X-ray absorptiometry (DXA) is the standard method for evaluating FNBMD, its high cost, limited accessibility, and associated radiation exposure restrict its widespread use in community screenings [7]. Consequently, developing a simple, cost-effective, and reliable alternative for routine FNBMD assessment is imperative.

Machine learning (ML), a branch of artificial intelligence (AI), excels at handling extensive heterogeneous datasets and capturing intricate relationships between features [8–10]. It has demonstrated significant potential in predicting healthcare outcomes and complications, aiding clinicians in making informed decisions, and improving patient care [11–13]. This opens innovative avenues for creating accurate and reliable models for the real-time prediction of FNBMD, which is crucial for assessing patients’ bone health and facilitating early osteoporosis diagnosis. Currently, ML algorithms have been effectively utilized to screen for osteoporosis with satisfactory outcomes [14–16]. Nevertheless, most studies have primarily focused on the models’ ability to identify patients with osteoporosis, rather than on the real-time prediction of bone mineral density (BMD) values. Moreover, while osteoporosis is more prevalent among women aged 50 and above, leading to a concentration of research within this demographic, fewer studies have addressed men of the same age group. There are increasing calls for routine BMD screenings for men aged 50 and older [17, 18]. Addressing this research gap, the application of ML algorithms for real-time FNBMD prediction in this male population holds substantial clinical significance.

The objective of this study is to develop and validate a straightforward, cost-effective, and reliable ML prediction model for FNBMD in elderly men. This involves comparing the predictive performance of various ML algorithms. The hypothesis of this study is that the optimal model can accurately predict FNBMD using only a limited set of readily accessible features. This capability is crucial for the early identification of osteoporosis in older men, enabling the implementation of targeted preventive strategies aimed at reducing the incidence of OFNFs.

## Materials and methods

**Figure 1.**
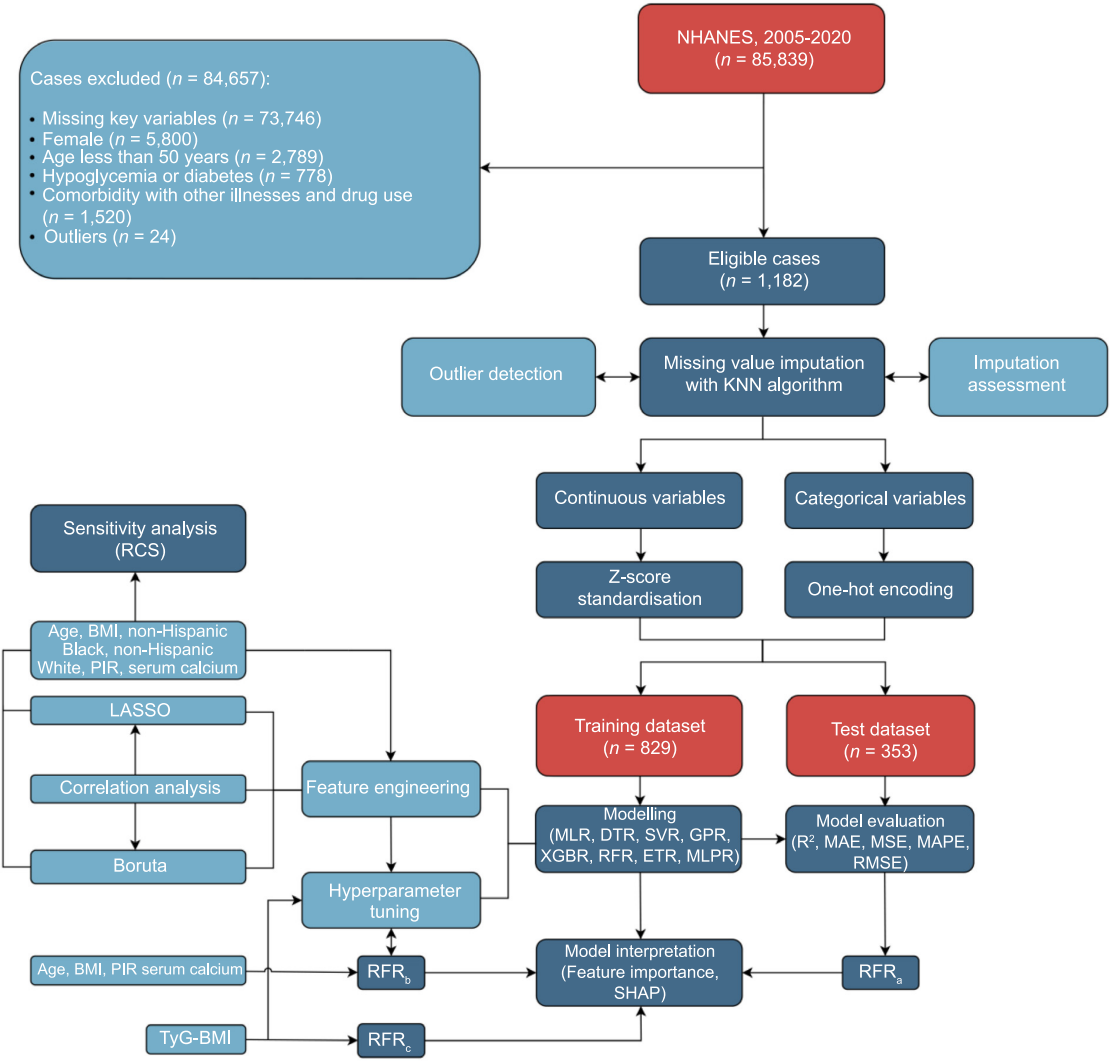
**Flowchart of the study design.** NHANES: National health and nutrition examination survey; KNN: K-nearest neighbors; MLR: Multivariable linear regression; DTR: Decision tree regression; SVR: Support vector regression; GPR: Gaussian process regression; XGBR: Extreme gradient boosting regression; RFR: Random forest regression; ETR: Extra-trees regression; MLPR: Multi-layer perceptron regression; R^2^: Coefficient of determination; MAE: Mean absolute error; MSE: Mean squared error; MAPE: Mean absolute percentage error; RMSE: Root mean squared error; TyG-BMI: Triglyceride and glucose-body mass index; PIR: Poverty income ratio; LASSO: Least absolute shrinkage and selection operator; RCS: Restricted cubic spline.

This study adhered to the Strengthening the Reporting of Observational Studies in Epidemiology (STROBE) reporting guidelines. The overall design is depicted in Figure 1.

### Data source

The data for this study were sourced from the continuous National Health and Nutrition Examination Survey (NHANES), conducted by the National Center for Health Statistics of the Centers for Disease Control and Prevention (CDC) (https://wwwn.cdc.gov/nchs/nhanes/). NHANES data were gathered from a nationally representative sample of the civilian, noninstitutionalized U.S. population, utilizing a multistage probability design. The dataset includes questionnaire responses, laboratory test results, and physical examination data.

### Study population

We incorporated data from NHANES covering the period from January 2005 to March 2020, excluding the 2011–2012 and 2015–2016 cycles due to the unavailability of FNBMD data. To ensure a comprehensive and representative dataset while avoiding potential biases and redundancies, we merged data from the specified periods and took careful measures to include each individual only once. The exclusion criteria were as follows: (1) female participants; (2) individuals under the age of 50; (3) participants with hypoglycemia (fasting plasma glucose [FPG] ≤ 50 mg/dL or 2.8 mmol/L) or diabetes (FPG ≥ 110 mg/dL or 7.0 mmol/L) at baseline; and (4) individuals with conditions including cancer, thyroid disorders, chronic renal failure, inflammatory arthritis, and chronic liver disease, as well as those using medications for dyslipidemia, corticosteroids, sex hormones, and diuretics. Participants with incomplete or invalid FNBMD data were excluded from the final analysis. Additionally, cases with outliers, defined as data points exceeding three times the interquartile range (IQR), were removed, as they constituted only 1% of the dataset.

### BMD examination

BMD measurements, expressed in grams per square centimeter (gm/cm^2^), were conducted using DXA with Hologic QDR 4500A fan-beam densitometers (Hologic Inc., Bedford, MA, USA) [1, 2]. All measurements were conducted by NHANES radiological technologists, who had undergone extensive training and certification. In the present research, FNBMD data were selected as the outcome because the femoral neck is often proposed as the reference skeletal site for defining osteoporosis in epidemiological research [3].

### Features

Based on the literature [4–6], the following features were included: age, body mass index (BMI), race, education level, marital status, drinking status, smoking status, physical activity, family history of osteoporosis (FHOS), poverty income ratio (PIR), systolic blood pressure (SBP), diastolic blood pressure (DBP), high-density lipoprotein (HDL), FPG, total cholesterol (TC), triglycerides, low-density lipoprotein (LDL), serum calcium, serum phosphorus, and serum 25-hydroxyvitamin D3 (25(OH)D3). Race was categorized as “Mexican American,” “non-Hispanic White,” “non-Hispanic Black,” or “other race”; education level was classified as “less than high school,” “high school,” or “college or above”; and marital status was divided into “married or living with partner” or “single.” Regarding health-related behaviors, smoking status was defined based on a lifetime history of smoking at least five packs of cigarettes (equivalent to 100 cigarettes), categorized as “yes” for those meeting this criterion and “no” for those who smoked fewer than five packs and were currently nonsmoking. Alcohol consumption was binary, with individuals drinking at least once a month over the past year classified as “yes” and all others as “no”. FHOS was specifically noted if a parent had been diagnosed with the condition. Physical activity was quantified in metabolic equivalent hours per week (MET-min/week) of moderate-to-vigorous physical activity, with categories set at “less active (< 600 MET-min/week)” and “active (≥ 600 MET-min/week)” [7].

### Missing data

The details of the missing data are presented in Table S1. To enhance statistical power and reduce bias, the K-nearest neighbor (KNN) imputation [19], with K equal to 10, was employed to address missing values in eligible cases. To assess the imputation effect, we performed separate outlier tests on the imputed data and compared them with the raw data to identify any between-group differences, as detailed in Table S1. Following the acquisition of the qualified interpolated data, we applied Z-score standardization to quantitative features and one-hot encoding to qualitative features. The data were then divided into a training set and a test set in a ratio of 7:3 for subsequent analysis.

### Feature selection

We implemented a stringent feature selection process to pinpoint the most pertinent predictors for constructing the prediction model, using only the training cohort to prevent data leakage. Initially, a pairwise Pearson or Spearman correlation matrix was utilized to evaluate the continuous features for collinearity, setting a correlation threshold of *r* > 0.8. Collinearity, which occurs when two or more predictor variables are highly correlated, can obscure the unique contribution of each variable to the outcome. Consequently, we selected the most readily available variables among the collinear ones for further analysis. Next, we employed a two-step approach using both the Boruta algorithm [8] and the least absolute shrinkage and selection operator (LASSO) [9]. We then took the intersection of the predictors identified by both algorithms to ensure only the most relevant and robust variables were included in the development of our prediction model. This combined methodology aims to enhance the model’s accuracy and generalizability while minimizing the risk of overfitting or incorporating irrelevant predictors.

### Model development and validation

Common supervised ML algorithms for regression encompass linear models such as linear regression, foundational tree-based methods like decision trees and ensemble-based random forests, support vector machines (SVMs) which excel in complex function mapping with kernel function selection, neural networks renowned for their adaptability and proficiency in managing noisy data, and Gaussian process models, prized for their probabilistic approach and ability to estimate prediction uncertainty [10]. (1) A linear model, specifically multivariable linear regression (MLR), posits a linear relationship between input and output variables. It is straightforward and establishes a baseline for comparison. While predicting phenomena such as FNBMD might surpass the complexity MLR can handle, it remains an excellent benchmark to gauge the degree of enhancement provided by more sophisticated non-linear ML algorithms. (2) Tree-based models such as decision tree regression (DTR), extreme gradient boosting regression (XGBR), random forest regression (RFR), and extra-trees regression (ETR) are highly effective in managing complex medical datasets. Renowned for their interpretability and capability to process both numerical and categorical data, these models excel at identifying non-linear relationships and intricate interactions among variables, making them particularly valuable in health research [11]. (3) Support vector regression (SVR), a variant of the SVM, is frequently selected for its effectiveness in high-dimensional spaces. SVR is particularly skilled at navigating the complex patterns prevalent in medical datasets, establishing it as a robust choice for intricate data analysis [12]. (4) Gaussian process regression (GPR), a non-parametric method, is highly valued for its ability to provide uncertainty measures alongside predictions. This feature is particularly advantageous for analyzing medical data, which often involves considerable uncertainty [13]. (5) Multi-layer perceptron regression (MLPR), a neural network approach, is adept at capturing the intricate and often non-linear patterns found in large health datasets [14]. By incorporating a diverse array of ML approaches, our study offers a comprehensive comparison of FNBMD prediction across various models.

All models were implemented using the “sklearn,” “xgboost,” “numpy,” and “pandas” packages, with grid search utilized to optimize the hyperparameters for each model. This method systematically explores a broad spectrum of hyperparameter values, enhancing the probability of discovering the most effective global solution for all critical parameters. This approach facilitates thorough yet efficient tuning, which is particularly advantageous given the limited size of our dataset. To assess the effectiveness of the predictive ML models, we employed five specific metrics for regression issues: the coefficient of determination (R^2^), mean absolute error (MAE), mean squared error (MSE), mean absolute percentage error (MAPE), and root mean squared error (RMSE).

### Model explainability

ML models often face challenges in terms of explainability, particularly as the complexity and accuracy of the models increase, potentially reducing interpretability [15, 16]. To address this issue, we utilized SHapley Additive exPlanations (SHAP) values from the game theory-based “shap” package to plot feature importance for global explainability. This approach enhanced our understanding of the decision-making processes within the model that demonstrated the best performance [16].

To ascertain the most critical features for predicting FNBMD, we employed the permutation importance method. This technique evaluates feature importance by assessing the impact of randomly permuting (shuffling) the values of a feature on the model’s predictive performance. To minimize error and stabilize the results, we conducted 1000 permutations for each feature across all constructed models, thereby generating 1000 importance values per feature. We then calculated the mean of these importance values and ranked the features based on these averages.

### Sensitivity analysis

To ensure the robustness of our main findings, we implemented three sensitivity analyses. Firstly, we removed racial features from the optimal model and then evaluated the new model’s performance in both the training and test sets. This was undertaken to develop a model independent of racial features, thereby expanding its applicability. Secondly, recognizing the documented reliability of the TyG-BMI index as a predictor of BMD [17, 18], we computed this index from imputed data and integrated it into the selected algorithm for comparison with our optimal model. Details on the TyG-BMI calculation are provided in the supplementary material (Data S1). Lastly, we conducted restricted cubic spline (RCS) analyses [20] for TyG-BMI and each quantitatively selected feature post-engineering, setting five knots at the 5th, 35th, 50th, 65th, and 95th percentiles to flexibly model its relationship with FNBMD. This analysis was aimed at verifying the plausibility of the interpretations suggested by the SHAP values.

### Sample size calculation

The R package “pmsampsize,” version 1.1.2, was employed to calculate the required minimum sample size for training [21]. We selected 22 candidate predictor parameters to construct a multivariable prediction model for the continuous outcome. We based our calculations on the assumption that an existing prediction model in the same field has an adjusted *R*^2^ of 0.8 [6, 17], and that FNBMD values in the present population have a mean of 0.81 and a standard deviation of 0.13. Consequently, the minimum sample size needed for the training cohort was determined to be 256 cases. Additionally, following Richard’s recommendations for external validation of a prognostic model [22], we used a confidence interval width of ≤ 5 for the calibration-in-the-large (considered precise on an outcome scale of 0–100) and ≤ 0.3 for the calibration slope to calculate the sample size, which indicated a minimum of 235 cases were required. This analysis confirms that the eligible population is adequate for both model construction and validation.

### Ethical statement

According to the Helsinki Declaration of 1975, as revised in 2000, all procedures adhered to the ethical standards of the responsible committee on human experimentation. All participants provided written informed consent to participate in this study, which was approved by the Institutional Review Board of the NHANES. All methods in this study were carried out in accordance with relevant guidelines and regulations.

### Statistical analysis

Continuous data were evaluated for normality using the Shapiro–Wilk test and presented as mean with standard deviation (SD) for normally distributed data, or median with IQR for non-normally distributed data. The outliers were detected using boxplots and Grubbs’ test. The homogeneity of variance across groups was assessed using the Levene test. For data following a Gaussian distribution, parametric tests such as the unpaired two-tailed Student’s *t*-test or Welch’s *t*-test were used for comparisons between the two groups. For non-Gaussian data, the Mann–Whitney *U* test was employed for two-group comparisons. Categorical data were expressed as counts and percentages and analyzed using the chi-squared test or Fisher’s exact test, the latter being applied when more than 20% of cells had expected frequencies of less than 5. Statistical significance was determined by a two-sided *P* value of less than 0.05. All statistical analyses were performed using R software, version 4.1.0.

## Results

### Participant characteristics

A total of 1182 eligible participants from the NHANES data collected between January 2005 and March 2020 were included in the analysis. The exclusion criteria are detailed in Figure 1. Missing values were found in BMI, smoking status, drinking status, and nearly all laboratory test-related features. Comparative analyses between the raw data and the imputed data showed no statistically significant differences (Table S1). No outliers were detected in either the complete dataset (data with all missing values removed) or the imputed data.

In the imputed dataset, the median age of participants was 61 years (IQR 55–68), and the median BMI was 33.4 kg/m^2^ (IQR 29.7–36.5). The majority were non-Hispanic White (42.8%), had at least a bachelor’s degree (48.5%), were not single (71.3%), and reported drinking alcohol (75.9%). The median FNBMD of these participants was 0.80 (IQR 0.71–0.89).

Of all eligible participants, 829 were assigned to the training group and 353 to the test group. Participant characteristics were similar across both cohorts, with no significant differences (*P* > 0.05) noted (Table 1). The median TyG-BMI values were 228 (IQR 200–260) for the training group and 231 (IQR 206–265) for the test group. The median FNBMD values were consistent across both groups, each registering at 0.80 (IQR 0.71–0.89).

**Table 1:** Baseline characteristics of training and test datasets

**Characteristics**	**Training set (*n* ═ 829)**	**Test set (*n* ═ 353)**	***P* value**
FNBMD (gm/cm^2^), median (IQR)	0.80 (0.71, 0.89)	0.80 (0.71, 0.89)	0.725
TyG-BMI, median (IQR)	228 (200, 260)	231 (206, 265)	0.135
Age (years), median (IQR)	61.0 (55.0, 68.0)	61.0 (55.0, 70.0)	0.225
BMI (kg/m^2^), median (IQR)	32.8 (29.7, 36.5)	33.4 (29.9, 36.5)	0.421
Race, *n* (%)			0.084
Mexican American	108 (13.0)	47 (13.3)	
Other Hispanic	93 (11.2)	25 (7.08)	
Non-Hispanic White	353 (42.6)	153 (43.3)	
Non-Hispanic Black	202 (24.4)	83 (23.5)	
Other race	73 (8.81)	45 (12.7)	
Education, *n* (%)			0.130
Less than high school	227 (27.4)	115 (32.6)	
High school	197 (23.8)	70 (19.8)	
College or above	405 (48.9)	168 (47.6)	
Marital status, *n* (%)			0.114
Married or living with partner	580 (70.0)	263 (74.5)	
Single	249 (30.0)	90 (25.5)	
Drinking status, *n* (%)			0.447
No	205 (24.7)	80 (22.7)	
Yes	624 (75.3)	273 (77.3)	
Smoking status, *n* (%)			0.292
No	571 (68.9)	254 (72.0)	
Yes	258 (31.1)	99 (28.0)	
Physical activity, *n* (%)			0.779
Less active	369 (44.5)	154 (43.6)	
Active	460 (55.5)	199 (56.4)	
FHOS, *n* (%)			0.367
No	765 (92.3)	331 (93.8)	
Yes	64 (7.72)	22 (6.23)	
PIR, median (IQR)	2.70 (1.40, 4.28)	2.59 (1.35, 4.04)	0.364
SBP (mmHg), median (IQR)	127 (117, 139)	128 (119, 139)	0.598
DBP (mmHg), median (IQR)	74.0 (67.0, 80.0)	74.0 (67.0, 81.0)	0.71
HDL (mmol/L), median (IQR)	1.29 (1.09, 1.55)	1.29 (1.09, 1.60)	0.751
FPG (mmol/L), median (IQR)	5.66 (5.33, 6.05)	5.77 (5.38, 6.11)	0.171
TC (mmol/L), median (IQR)	4.99 (4.40, 5.61)	4.97 (4.32, 5.56)	0.323
Triglycerides (mmol/L), median (IQR)	1.09 (0.81, 1.43)	1.09 (0.79, 1.46)	0.522
LDL (mmol/L), median (IQR)	3.10 (2.53, 3.60)	3.03 (2.46, 3.60)	0.346
Serum calcium (mmol/L), median (IQR)	2.33 (2.28, 2.38)	2.33 (2.28, 2.38)	0.609
Serum phosphorus (mmol/L), median (IQR)	1.10 (1.00, 1.20)	1.07 (0.97, 1.16)	0.565
Serum 25(OH)D3 (ng/mL), median (IQR)	59.7 (52.4, 67.6)	58.9 (51.0, 67.0)	0.253

*P* values between groups were assessed by the chi-square and Mann–Whitney *U* tests. IQR: Inter-quartile range; FNBMD: Femoral neck bone mineral density; TyG-BMI: Triglyceride and glucose to body mass index; FHOS: Family history of osteoporosis; PIR: Poverty income ratio; SBP: Systolic blood pressure; DBP: Diastolic blood pressure; HDL: High-density lipoprotein; FPG: Fasting plasma glucose; TC: Total cholesterol; LDL: Low-density lipoprotein.

### Feature selection

**Figure 2.**
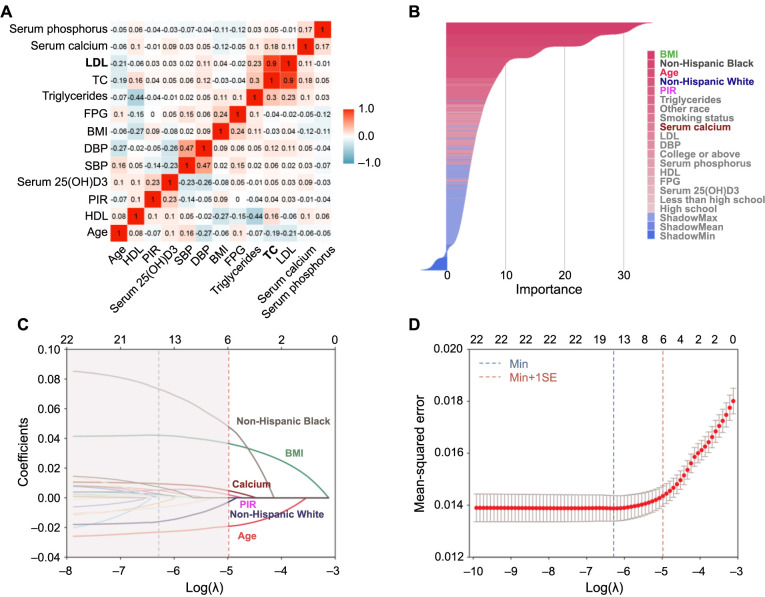
**Feature engineering.** (A) Displaying Spearman and Pearson correlation matrix of continuous variables. Feature pairs with correlation coefficients greater than 0.8 are bolded; (B) Illustrating feature selection with the Boruta algorithm. The features highlighted in bold and color are identified by intersecting the results from the Boruta and LASSO algorithms; (C and D) Showcasing feature selection using the LASSO algorithm. HDL: High-density lipoprotein; PIR: Poverty income ratio; SBP: Systolic blood pressure; DBP: Diastolic blood pressure; BMI: Body mass index; FPG: Fasting plasma glucose; TC: Total cholesterol; LDL: Low-density lipoprotein.

As depicted in Figure 2A, Pearson correlation values for LDL and TC exceeded 0.8, signaling the presence of collinearity. We opted to select LDL for the subsequent feature selection phase. The Boruta algorithm (Figure 2B) and LASSO algorithm (Figure 2C and 2D) identified a total of six features as significant predictors of the outcome. These encompassed age, BMI, non-Hispanic Black, non-Hispanic White, PIR, and serum calcium. The chosen features were then integrated into eight ML regressors to develop predictive models.

### Hyperparameters tuning

Table S2 lists the optimized hyperparameters for each algorithm. Hyperparameter tuning was not conducted on the MLR model, as it does not involve hyperparameters.

### Development and validation of prediction models

The identified six predictors and optimized hyperparameters were incorporated into the FNBMD prediction regressors. Within the training cohort, the RFR model demonstrated superior performance, achieving the highest *R*^2^ at 0.712, the lowest MSE at 0.005, and an RMSE of 0.072. Upon removing the racial features from the model, the performance of the adjusted model (referred to as ABPC-RFR, which includes age, BMI, PIR, and serum calcium) improved significantly, with *R*^2^ rising to 0.841 and reductions in MAE, MSE, MAPE, and RMSE reaching the most favorable values of 0.043, 0.003, 0.054, and 0.057, respectively. This model outperformed all others, including the TyG-BMI-RFR model, which solely incorporates TyG-BMI. In the test cohort, the ABPC-RFR model’s performance was comparable to that of the original RFR model, which integrates six features, and it surpassed the performance of all other models examined (as detailed in Table 2).

**Table 2:** FNBMD prediction results for each model with metrics

**Model**	***R* ^2^**		**MAE**		**MSE**		**MAPE**		**RMSE**	
	**Training**	**Test**	**Training**	**Test**	**Training**	**Test**	**Training**	**Test**	**Training**	**Test**
MLR	0.264	0.168	0.092	0.095	0.014	0.014	0.116	0.121	0.116	0.120
DTR	0.252	0.150	0.092	0.095	0.014	0.015	0.117	0.121	0.117	0.121
SVR	0.298	0.204	0.090	0.093	0.013	0.014	0.114	0.119	0.114	0.117
GPR	0.691	0.099	0.058	0.106	0.006	0.019	0.072	0.134	0.075	0.138
XGBR	0.174	0.115	0.096	0.097	0.015	0.015	0.119	0.123	0.123	0.123
RFR^a^	0.712	**0.218**	0.053	**0.092**	0.005	**0.013**	0.067	**0.118**	0.072	**0.116**
RFR^b^	**0.821**	0.199	**0.043**	**0.092**	**0.003**	0.014	**0.054**	0.119	**0.057**	0.117
RFR^c^	0.158	0.073	0.097	0.100	0.015	0.017	0.124	0.127	0.123	0.130
ETR	0.708	0.212	0.052	0.093	0.005	0.014	0.064	**0.118**	0.073	0.117
MLPR	0.135	0.094	0.098	0.097	0.016	0.016	0.119	0.119	0.126	0.125

The metrics of the best-performing model are bolded. ^a^The model included predictors of age, BMI, PIR, non-Hispanic Black, non-Hispanic White, and serum calcium. ^b^The model included predictors of age, BMI, PIR, and serum calcium. ^c^The model employed TyG-BMI as a predictor. FNBMD: Femoral neck bone mineral density; R^2^: Coefficient of determination; MAE: Mean absolute error; MSE: Mean squared error; MAPE: Mean absolute percentage error; RMSE: Root mean squared error; MLR: Multivariable linear regression; DTR: Decision tree regression; SVR: Support vector regression; GPR: Gaussian process regression; XGBR: Extreme gradient boosting regression; RFR: Random forest regression; ETR: Extra-trees regression; MLPR: Multi-layer perceptron regression; TyG-BMI: Triglyceride and glucose-body mass index.

**Figure 3.**
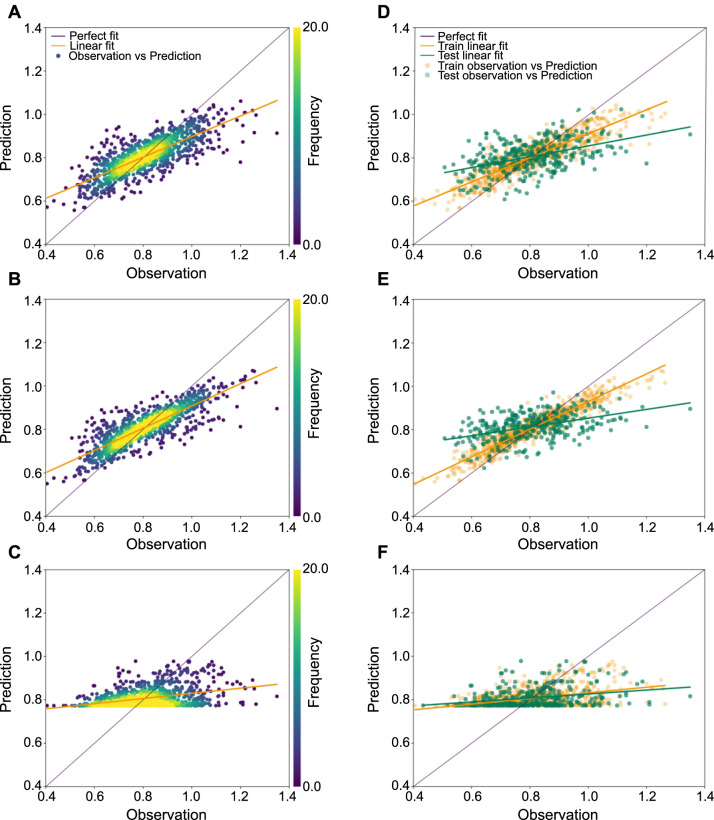
**Scatter plots for FNBMD prediction in RFR models.** (A and D) Illustrating scatter density plots (A) and scatter training/test plots (D) for the RFR model, incorporating age, BMI, non-Hispanic Black and White, PIR, and serum calcium; (B and E) Displaying plots for the RFR model including age, BMI, PIR, and serum calcium; (C and F) Displaying plots for the RFR model solely containing the TyG-BMI index. Scatter density plots comprise all data points, whereas the scatter plots are separately composed of training and test sets. FNBMD: Femoral neck bone mineral density; RFR: Random forest regressor; BMI: Body mass index; PIR: Poverty income ratio; TyG-BMI: Triglyceride and glucose-body mass index.

In Figure 3, the predictive behavior of the RFR, ABPC-RFR, and TyG-BMI-RFR models varies with the true value of FNBMD. Specifically, when the true FNBMD value is below 0.8, the predicted values from these models are consistently higher than the actual values. Conversely, when the true FNBMD value exceeds 0.8, the predictions fall below the true values. This pattern suggests a decline in model performance when predicting extreme values. The scatter density plots (Figure 3A, 3B, and 3C) show that the ABPC-RFR model’s fitted line deviates the least from the ideal line, signifying it has the best predictive performance among the models. Similarly, in the scatter plots for the training set (Figure 3D, 3E, and 3F), the ABPC-RFR model again shows the smallest angle of deviation from the perfect line, indicating superior performance in the training phase. In the test set, the fitted lines of the RFR and ABPC-RFR models exhibit similar angles of deviation from the perfect line, suggesting that their performance in the test set is comparable. These observations align with the findings presented in Table 2.

### Model explainability

**Figure 4.**
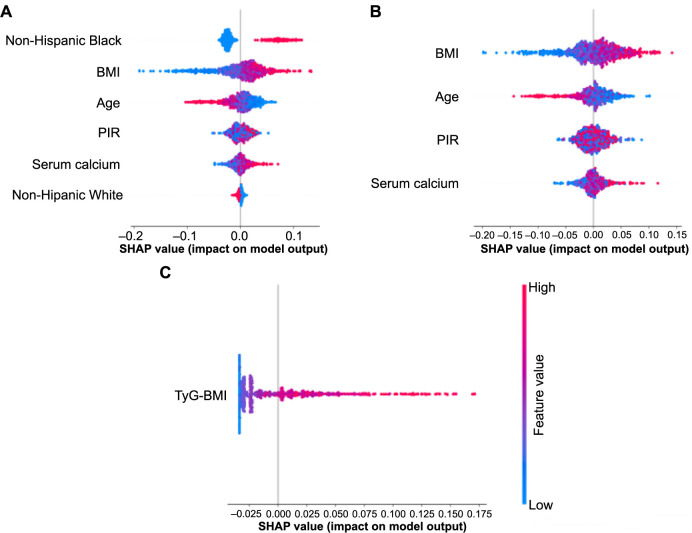
**SHAP value of features and their impact on the model output for FNBMD prediction in RFR models.** (A) Displaying SHAP plots for the RFR model, incorporating age, BMI, non-Hispanic Black and White, PIR, and serum calcium; (B) Illustrating SHAP plots for the RFR model including age, BMI, PIR, and serum calcium; (C) Showcasing SHAP plots for the RFR model solely containing the TyG-BMI index. SHAP: Shapley additive exPlanations; FNBMD: Femoral neck bone mineral density; BMI: Body mass index; PIR: Poverty income ratio; TyG-BMI: Triglyceride and glucose-body mass index.

**Figure 5.**
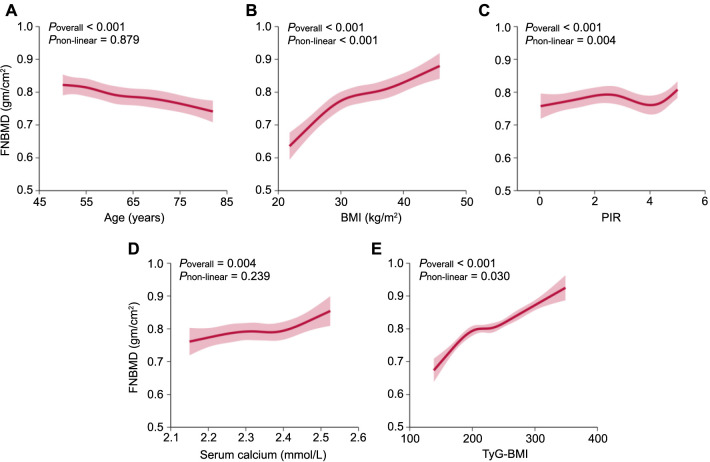
**Restricted cubic spline analysis.** (A–E) Displaying the association between continuous features and FNBMD. Each curve employs five knots located at the 5th, 35th, 50th, 65th, and 95th percentiles. Shaded regions denote the 95% confidence intervals. FNBMD: Femoral neck bone mineral density; BMI: Body mass index; PIR: Poverty income ratio; TyG-BMI: Triglyceride and glucose-body mass index.

The SHAP summary plots illustrate the impact of each feature across the random forest (RF) model (Figure 4A), the adjusted BMI, PIR, and serum calcium random forest (ABPC-RFR) model (Figure 4B), and the TyG-BMI random forest (TyG-BMI-RFR) model (Figure 4C). SHAP values above zero suggest higher FNBMD values, whereas values below zero indicate lower FNBMD values. For instance, a higher BMI (depicted in red) correlates with larger SHAP values, suggesting that individuals with a higher BMI tend to have greater FNBMD values. Conversely, as age increases, the SHAP values typically decrease, indicating an association between advancing age and bone loss. These trends are depicted in Figure 5A and 5B. Additionally, a higher TyG-BMI value generally results in a larger SHAP value, demonstrating a positive relationship between this index and FNBMD (Figure 4C).

Permutation feature importance analysis identified the pivotal features for predicting FNBMD. To evaluate the relative importance of these features across all eight models, a ranking score system was employed. The most critical feature in each model was assigned a ranking of 1, with rankings progressively decreasing down to 6 for the least significant variable. As detailed in Table 3, BMI emerged as the most influential predictor, achieving a mean ranking of 1.0 ± 0.0 and a median ranking of 1.0 (IQR 1.0–1.0). It was followed by non-Hispanic Black, age, PIR, serum calcium, and non-Hispanic White in terms of their impact on FNBMD prediction.

### Sensitivity analysis

In the RCS analysis depicted in Figure 5, age demonstrates a negative linear correlation with FNBMD, as evidenced by non-significance for non-linearity (*P* ═ 0.879) (Figure 5A). Serum calcium displays a positive linear relationship with FNBMD (*P* for non-linearity ═ 0.239) (Figure 5D). BMI and TyG-BMI both exhibit positive non-linear associations with FNBMD, with *P* values for non-linearity being less than 0.001 and 0.030, respectively (Figure 5B and Figure 5E). PIR also shows a distinct non-linear association with FNBMD (*P* for non-linearity < 0.001) (Figure 5C). These findings align with the interpretations provided by the SHAP values, confirming the consistency of the results across different analytical approaches.

## Discussion

Osteoporotic fractures of the proximal femur significantly impact health and increase the mortality rate among the elderly. Early detection of osteoporosis and the initiation of anti-osteoporotic treatments are critical in preventing such fractures. FNBMD is utilized both for diagnosing osteoporosis and as a key predictor of OFNFs. However, the methods currently available for assessing FNBMD are not suitable for routine osteoporosis screening. Therefore, it is essential to develop a straightforward, cost-effective, and reliable alternative method for assessment. In this study, we created and validated an ML-based prediction model for FNBMD specifically for men aged 50 years and older. Through meticulous feature selection, six key predictors were identified: age, BMI, non-Hispanic Black, non-Hispanic White, PIR, and serum calcium. Among the eight models evaluated, the RFR model showed the best performance across all metrics. When race-related variables were excluded, the adjusted RFR model (ABPC-RFR), incorporating the remaining predictors, performed comparably to the full RFR model and significantly outperformed the TyG-BMI-RFR model. The simplified model required only three demographic factors (age, BMI, and PIR) and one laboratory test variable (serum calcium) for making predictions. This simplicity enhances its usability for routine osteoporosis checks among older men of diverse races, aligning well with the study’s hypothesis.

Our important analysis revealed that BMI was the most valuable predictor of FNBMD in this study population (Table 3). Indeed, BMI is not only a simple and widely used health indicator but also a significant predictor of bone tissue structure, closely associated with BMD [23–25]. Compared to the BMD of the lumbar vertebral body, FNBMD is more stable and less influenced by factors such as degeneration, osteophytes, and sclerosis [26]. Several studies have consistently shown a positive correlation between BMI and the absolute values of FNBMD, exhibiting a synergistic increasing trend [19, 23–25]. In research involving 900 elderly individuals, Dogan et al. [27] confirmed that femoral BMD increased with rising BMI levels, noting statistically significant differences in femoral BMD among men across different BMI categories, with the highest levels observed in the obese group (BMI of 30 kg/m^2^ and above) compared to those with ideal body weight. Similarly, Kirchengast et al. [28] found that BMD increased with both weight and BMI in both sexes, with higher BMD values in overweight individuals compared to those with ideal body weight, aligning with the findings of our study (Figure 4A and 4B and 5B).

By combining model interpretative analysis with RCS analysis, we observed that changes in FNBMD values with increasing BMI were more pronounced when BMI was below 30 and diminished when BMI exceeded 30. This pattern may be attributed to both mechanical effects—where heavier loads on the skeleton induce bone-specific deformations that stimulate osteoblast activity, enhancing the synthesis and expression of osteoblast-related genes, thereby increasing bone density and enabling the skeleton to adapt to applied stress [29, 30]—and hormonal effects due to increased estrogen production in adipose tissue. Weight gain and enhanced adipose tissue may promote the conversion of androgens to estrogens, improving bone mass in both men and women while maintaining healthy levels of insulin and regulatory factors like insulin-like growth factor-1, leptin, and lipocalin [26, 31]. These observations suggest potential variations in FNBMD between non-obese and obese populations, possibly necessitating distinct diagnostic criteria for osteoporosis in each group. Additionally, to address the modifiable factor of BMI, we recommend promoting exercise, particularly strength training, to increase lean body mass and stimulate bone remodeling to better accommodate loading. However, it is crucial to note that while a higher BMI can be protective against osteoporosis, maintaining a BMI around or slightly below 30 is advisable to prevent the increased risks of falls, degenerative changes, and systemic diseases associated with obesity in the elderly [26, 27].

Age is another important predictor of FNBMD. In this research, we used SHAP values to understand the ML models (RFR and ABPC-RFR) along with RCS analysis to show how age and FNBMD are related. The results showed that age is inversely related to FNBMD, i.e., BMD decreases significantly with age. This finding is consistent with the existing literature [25, 26]. In a study of adult men in Kosovo, Hoxha et al. [25] identified a negative relationship between age and FNBMD, attributing this natural bone mass loss to the aging process. This insight is crucial for guiding local BMD assessments and initiating early preventive strategies against osteoporosis and related fractures in older men. Similarly, Jiang et al. [26] studied the FNBMD of 358 Chinese males aged 50 and older, arriving at the same conclusion. They interpreted this trend as a decline in bone mass beginning around the age of 50, due to osteoblast dysfunction and an increase in osteoclast resorption. The application of ML in this research offers a detailed understanding of how age interacts with other factors to influence bone health. Importantly, incorporating age as a predictor in our model underscores the necessity of early intervention and tailored preventive measures for the elderly. The data-driven insights provided by this study showcase the potential of ML to enhance predictive models for FNBMD, enabling healthcare providers to more effectively identify and manage individuals at high risk for osteoporosis.

The association between PIR and FNBMD observed in our study adds a critical socioeconomic dimension to the understanding of bone health. Our analysis revealed that a higher PIR (≥ 4 in the RCS analysis), indicative of greater socioeconomic status, correlates with improved FNBMD. This aligns with research by Du et al. [32], who reported similar findings and suggested that individuals with higher socioeconomic status have better access to healthcare resources, nutrition, and lifestyle choices conducive to bone health. A meta-analysis encompassing eight epidemiological studies demonstrated that most population-based research supports the observation that individuals with higher income levels are more likely to exhibit higher BMD [33]. This finding was further validated in another cross-sectional study involving 11,075 representative participants from the United States [34]. The positive impact of socioeconomic status on FNBMD emphasizes the potential barriers faced by lower-income populations in maintaining bone health, possibly due to limited access to nutritious food, healthcare services, and opportunities for physical activity [34]. Addressing these disparities could significantly improve bone health outcomes across different population segments. Therefore, our findings advocate for targeted public health interventions and policies that enhance access to bone health resources in economically disadvantaged communities, potentially reducing the prevalence of osteoporosis-related complications.

**Table 3:** Predictor importance ranking in each model

**Model**	**BMI**		**Non-Hispanic Black**		**Age**		**PIR**		**Serum calcium**		**Non-Hispanic White**	
	**Training**	**Test**	**Training**	**Test**	**Training**	**Test**	**Training**	**Test**	**Training**	**Test**	**Training**	**Test**
MLR	1	1	2	2	3	3	6	4	4	5	5	6
DTR	1	1	2	2	3	3	4	4	5	5	6	6
SVR	1	1	2	2	3	3	5	4	4	5	6	6
GPR	1	1	6	5	2	2	4	3	3	6	5	4
XGBR	1	1	3	2	2	3	5	5	6	6	4	4
RFR	1	1	2	2	3	3	4	4	5	5	6	6
ETR	1	1	2	2	3	3	4	4	5	5	6	6
MLPR	1	1	2	3	3	4	4	2	6	6	5	5
Mean ± SD	1.0 ± 0.0	1.0 ± 0.0	2.6 ± 1.4	2.5 ± 1.1	2.8 ± 0.5	3.0 ± 0.5	4.5 ± 0.8	3.8 ± 0.9	4.8 ± 1.0	5.4 ± 0.5	5.4 ± 0.7	5.4 ± 0.9
Median (IQR)	1.0 (1.0, 1.0)	1.0 (1.0, 1.0)	2.0 (2.0, 2.3)	2.0 (2.0, 2.3)	3.0 (2.8, 3.0)	3.0 (3.0, 3.0)	4.0 (4.0, 5.0)	4.0 (3.8, 4.0)	5.0 (4.0, 5.3)	5.0 (5.0, 6.0)	5.5 (5.0, 6.0)	6.0 (4.8, 6.0)

BMI: Body mass index; PIR: Family income to poverty ratio; MLR: Multivariable linear regression; DTR: Decision tree regression; SVR: Support vector regression; GPR: Gaussian process regression; XGBR: Extreme gradient boosting regression; RFR: Random forest regression; ETR: Extra-trees regression; MLPR: Multi-layer perceptron regression; SD: Standard deviation; IQR: Inter-quartile range.

Serum calcium, a critical element in bone metabolism, was positively correlated with FNBMD in our study, supporting the hypothesis that adequate calcium levels are essential for optimal bone density. This observation is in concordance with the work of Pan et al. [35], who noted that calcium plays a pivotal role not only in bone formation but also in maintaining the structural integrity of the bone matrix. The implications of these findings suggest that monitoring and managing serum calcium levels could be a key strategy for preventing bone density deterioration, especially in populations at risk for osteoporosis [36]. Additionally, our models reinforce the importance of integrating nutritional and metabolic factors into comprehensive assessments of bone health, advocating for a holistic approach to osteoporosis prevention and treatment strategies that encompass dietary calcium intake and its metabolic management.

The TyG-BMI, a novel metabolic marker explored in our study, demonstrated a significant predictive value for FNBMD. This finding underscores the intertwined roles of metabolic health and bone density. Notably, our analysis indicates that higher TyG-BMI values correlate with increased FNBMD, suggesting that metabolic efficiency and body composition collectively influence bone health. This relationship mirrors the results presented by Zhan et al. [37], who found that metabolic markers like the TyG index provide insight into the risk of metabolic bone diseases beyond traditional lipid and glucose measurements. The association between TyG-BMI and FNBMD enhances our understanding of how composite indices, which encapsulate multiple metabolic risks, can serve as effective tools for assessing bone health. Emphasizing the TyG-BMI in clinical evaluations could offer a more comprehensive assessment strategy, aiding in the early identification of individuals at risk for osteoporosis, thus facilitating timely intervention strategies.

Leveraging demographic (age, BMI, and PIR), laboratory test (serum calcium) characteristics, and the novel metabolic index (TyG-BMI) associated with FNBMD, we selected the RFR algorithm to construct three models: the RFR, ABPC-RFR, and TyG-BMI-RFR model. Among these, the ABPC-RFR model emerged as the simplest and most practical. The computation of TyG-BMI is relatively cumbersome, and the predictive efficacy based on this index is significantly lower compared to the ABPC-RFR model. Furthermore, the fact that race-related characteristics do not influence the ABPC-RFR model’s performance suggests that race may not be a prominent factor in our specific dataset. These findings indicate that the ABPC-RFR model can effectively predict FNBMD in men over 50 years of age and holds potential for routine community screening. This facilitates the early detection of osteoporosis and the initiation of anti-osteoporosis treatments to prevent OFNFs. It is noteworthy that the present study did not encompass races from Asia and other regions. This limitation could influence the generalizability of our findings. Future analyses should aim to include more diverse racial groups to better understand the impact of race on FNBMD predictions and ensure the model’s applicability across different populations.

Our study has several strengths. First, we utilized the large, nationally representative NHANES dataset, which provides a diverse and statistically significant sample, enhancing the generalizability of our findings. Meticulous data processing techniques were also employed, including the handling of outliers and the KNN estimation of missing data, ensuring data integrity. Furthermore, our study featured rigorous feature selection and a comprehensive model development and validation process. Multiple ML algorithms were utilized to pinpoint the best-performing model, optimizing predictive accuracy. Moreover, we incorporated various evaluation metrics and model interpretability techniques, such as SHAP values, to ensure transparency and facilitate the interpretation of results. Lastly, several sensitivity analyses were conducted to test the robustness of our findings, ensuring the reliability of our conclusions in different scenarios and settings.

The present study has several limitations that warrant consideration. First, the exclusion of female participants and certain age groups limits the generalizability of our findings to the entire population. Women, particularly post-menopausal women, are at high risk for osteoporosis, and the dynamics of BMD may differ significantly between genders. Our study also excluded patients with hypoglycemia, diabetes, and those on medications for chronic diseases. These exclusions were made to control for potential confounding factors that could introduce bias into the model. However, we recognize that these patients represent a significant subset of the population, and their exclusion may limit the applicability of our findings to broader populations. Future research should aim to validate our models in more diverse populations that include these important subsets to ensure the generalizability and robustness of the predictive models and to detect changes in the ranking of feature importance. Additionally, our reliance on cross-sectional data from NHANES prevents us from establishing causality between the predictors and FNBMD; longitudinal studies would be required to confirm the directionality of these associations. Another limitation is the potential for residual confounding due to variables not included or not available in the NHANES dataset. Lastly, while ML models offer potent predictive capabilities, generalizing these models to different healthcare settings can be challenging. Future research should focus on further validating the model across diverse populations to ensure its broader applicability and effectiveness in varying healthcare contexts.

## Conclusion

In conclusion, our study demonstrates that FNBMD can be easily and accurately assessed by integrating four readily accessible features (age, BMI, PIR, and serum calcium) through advanced ML techniques, outperforming the novel metabolic index (TyG-BMI). Our model interpretation results provide a deeper and more nuanced understanding of femoral neck bone health risks. These insights pave the way for more targeted and effective interventions to prevent and manage OFNFs in the development of femoral neck osteoporosis, especially in populations traditionally underrepresented in bone health studies, such as older men. Future research should aim to validate these predictive models in a broader range of populations to enhance their applicability and effectiveness. By continually refining these models and addressing the limitations outlined, we can better meet the healthcare needs of diverse populations and improve outcomes in bone health management.

## Supplemental data

**Data S1. Triglyceride and glucose-body mass index (TyG-BMI)**

Body mass index (BMI) was calculated using the weight divided by the square of height (weight/height^2^) formula. After an overnight fast, venous samples were collected using aseptic techniques for the analysis of phosphorus, total calcium, plasma glucose, and lipid levels.

The definitions of the TyG-BMI terms were determined as follows:

1. The TyG index was calculated using the formula: Ln [triglycerides (TG) in mg/dL × fasting plasma glucose (FPG) in mg/dL / 2].
2. TyG-BMI was derived by multiplying the TyG index by BMI, expressed in kg/m^2^.

**Table S1:** Comparisons between raw and imputation datasets

**Characteristics**	**Missing values** * n* **(%)**	**Raw dataset** **(*n* ═ 1182)**	**Imputation dataset** **(*n* ═ 1182)**	*P* **value**
FNBMD (gm/cm^2^), median (IQR)		0.80 (0.71, 0.89)	0.80 (0.71, 0.89)	1.000
Age (years), median (IQR)		61.0 (55.0, 68.0)	61.0 (55.0, 68.0)	1.000
BMI (kg/m^2^), median (IQR)	20 (1.7)	32.8 (29.7, 36.6)	33.4 (29.7, 36.5)	0.917
Race, *n* (%)				1.000
Mexican American		155 (13.1)	155 (13.1)	
Other Hispanic		118 (10.0)	118 (10.0)	
Non-Hispanic White		506 (42.8)	506 (42.8)	
Non-Hispanic Black		285 (24.1)	285 (24.1)	
Other race		118 (10.0)	118 (10.0)	
Education, *n* (%)				1.000
Less than high school		342 (28.9)	342 (28.9)	
High school		267 (22.6)	267 (22.6)	
College or above		573 (48.5)	573 (48.5)	
Marital status, *n* (%)				1.000
Married or living with partner		843 (71.3)	843 (71.3)	
Single		339 (28.7)	339 (28.7)	
Drinking status, *n* (%)	276 (23.4)			0.365
No		235 (25.9)	285 (24.1)	
Yes		671 (74.1)	897 (75.9)	
Smoking status, *n* (%)	426 (36.0)			0.056
No		487 (64.4)	825 (69.8)	
Yes		269 (35.6)	357 (30.2)	
Physical activity, *n* (%)				1.000
Less active		523 (44.2)	523 (44.2)	
Active		659 (55.8)	659 (55.8)	
FHOS, *n* (%)				1.000
No		1096 (92.7)	1096 (92.7)	
Yes		86 (7.28)	86 (7.28)	
PIR, median (IQR)	118 (10.0)	2.44 (1.29, 4.42)	2.64 (1.39, 4.21)	0.355
SBP (mmHg), median (IQR)		127.0 (117.0, 140.0)	127.0 (117.0, 139.0)	0.745
DBP (mmHg), median (IQR)	70 (5.9)	74.0 (67.0, 81.0)	74.0 (67.0, 80.8)	0.979
HDL (mmol/L), median (IQR)	24 (2.0)	1.29 (1.09, 1.55)	1.29 (1.09, 1.55)	0.670
FPG (mmol/L), median (IQR)	4 (0.3)	5.72 (5.33, 6.11)	5.72 (5.33, 6.11)	0.947
TC (mmol/L), median (IQR)	(1.4)	4.97 (4.37, 5.61)	4.97 (4.37, 5.61)	0.847
Triglycerides (mmol/L), median (IQR)	67 (5.7)	1.07 (0.79, 1.45)	1.09 (0.80, 1.44)	0.404
LDL (mmol/L), median (IQR)	21 (1.8)	3.08 (2.51, 3.60)	3.08 (2.51, 3.60)	0.764
Serum calcium (mmol/L), median (IQR)	28 (2.4)	2.33 (2.28, 2.38)	2.33 (2.28, 2.38)	0.985
Serum phosphorus (mmol/L), median (IQR)	14 (1.2)	1.07 (1.00, 1.20)	1.07 (1.00, 1.18)	0.986
Serum 25(OH)D3 (ng/mL), median (IQR)	668 (56.5)	59.3 (42.0, 75.1)	59.4 (51.9, 67.5)	0.545

*P* values between groups were assessed by the chi-square and Mann–Whitney U tests. IQR: Inter-quartile range; FNBMD: Femoral neck bone mineral density; BMI: Body mass index; FHOS: Family history of osteoporosis; PIR: Poverty income ratio; SBP: Systolic blood pressure; DBP: Diastolic blood pressure; HDL: High-density lipoprotein; FPG: Fasting plasma glucose; TC: Total cholesterol; LDL: Low-density lipoprotein.

**Table S2:** Optimization of hyperparameters using grid search

**Model**	**Hyperparameter**	**Range of hyperparameter in grid search**	**Selected value**
MLR	–	–	–
DTR	max_depth	50, 200, 500, 800, 1000, 1200	1000
	min_samples_split	2, 10, 18, 26, 34, 42	10
	min_samples_leaf	3, 11, 19, 27, 35, 43	43
	max_features	0.8, 0.9, 1.0	0.8
SVR	gamma	10^-6^, 10^-5^, 10^-4^, 10^-3^, 10^-2^, 10^-1^, 10^0^	10^-1^
	C	10^-3^, 10^-2^, 10^-1^, 10^0^, 10^1^, 10^2^, 10^3^, 10^4^	10^-1^
	epsilon	10^-3^, 10^-2^, 10^-1^, 10^0^, 10^1^, 10^2^	10^-1^
GPR	alpha	10^-10^, 10^-9^, 10^-8^, 10^-7^, 10^-6^, 10^-5^, 10^-4^, 10^-3^, 10^-2^, 10^-1^, 10^0^, 10^1^	10^-1^
XGBR	n_estimators	100, 200, 400	100
	max_depth	3, 5, 7, 10	7
	learning_rate	0.1, 0.2, 0.3	0.3
	gamma	0, 5, 10, 20	0
	reg_alpha	5, 10, 15	5
RFR^a^	n_estimators	100, 200, 400	100
	criterion	‘squared_error’, ‘absolute_error’, ‘poisson’	‘absolute_error’
	max_depth	10, 50, 100, 200, 400	100
	min_samples_split	2, 4, 8, 16	2
	min_samples_leaf	1, 2, 4, 8	2
	max_features	0.8, 0.9, 1.0	0.8
RFR^b^	n_estimators	100, 200, 400	200
	criterion	‘squared_error’, ‘absolute_error’, ‘poisson’	‘absolute_error’
	max_depth	10, 50, 100, 200, 400	400
	min_samples_split	2, 4, 8, 16	4
	min_samples_leaf	1, 2, 4, 8	1
	max_features	0.8, 0.9, 1.0	0.9
RFR^c^	n_estimators	100, 200, 400	100
	criterion	‘squared_error’, ‘absolute_error’, ‘poisson’	‘poisson’
	max_depth	10, 50, 100, 200, 400	10
	min_samples_split	2, 4, 8, 16	8
	min_samples_leaf	1, 2, 4, 8	2
	max_features	0.8, 0.9, 1.0	0.9
ETR	n_estimators	100, 200, 400	200
	criterion	‘squared_error’, ‘absolute_error’	‘absolute_error’
	max_depth	10, 50, 100	100
	min_samples_split	2, 4, 8, 16	2
	min_samples_leaf	1, 2, 4, 8	1
	max_features	0.6, 0.8, 1.0	0.8
	max_samples	0.8, 0.9, 1.0	1.0
	ccp_alpha	0, 1, 10, 100, 1000	0
MLPR	hidden_layer_sizes	(10,), (20,), (10, 10), (20, 20), (10, 20, 10)	(10, 10)
	activation	‘identity’, ‘tanh’, ‘relu’	‘identity’
	solver	‘sgd’, ‘adam’	‘adam’
	alpha	10^-4^, 10^-3^, 10^-2^, 10^-1^	10^-2^
	learning_rate	‘constant’, ‘adaptive’	‘constant’
	learning_rate_int	10^-3^, 10^-2^, 10^-1^	10^-1^

^a^The model included predictors of age, BMI, PIR, non-Hispanic Black, non-Hispanic White, and serum calcium. ^b^The model included predictors of age, BMI, PIR, and serum calcium. ^c^The model employed TyG-BMI as a predictor. MLR: Multivariable linear regression; DTR: Decision tree regression; SVR: Support vector regression; GPR: Gaussian process regression; XGBR: Extreme gradient boosting regression; RFR: Random forest regression; ETR: Extra-trees regression; MLPR: Multi-layer perceptron regression; TyG-BMI: Triglyceride and glucose-body mass index.

## Data Availability

The datasets analyzed during the current study are available on the NHANES website: https://www.cdc.gov/nchs/index.htm.
